# Two-dimensional infrared spectroscopic study of cytochrome *c* peroxidase activity in deep eutectic solvent

**DOI:** 10.1063/1.5130940

**Published:** 2019-12-18

**Authors:** Koji Osawa, Dorota Kossowska, Kwanghee Park, Kyungwon Kwak, Minhaeng Cho

**Affiliations:** 1 Center for Molecular Spectroscopy and Dynamics, Institute for Basic Science (IBS), Seoul 02841, South Korea; 2 Department of Chemistry, Korea University, Seoul 02841, South Korea

## Abstract

Deep eutectic solvents (DESs) prepared by mixing hydrogen-bond donor and acceptor molecules have been found to be of use in several applications. Recently, it was shown that DESs can enhance the peroxidation activity of cytochrome *c*. Here, to elucidate the effects of DESs on the peroxidase activity of cytochrome *c*, we carried out linear and nonlinear infrared spectroscopic studies of the CO stretch mode of carbon monoxide cytochrome *c* (COCyt*c*) in ethylammonium chloride (EAC)/urea DES. The FTIR spectrum of COCyt*c* shows a significant spectral shift upon addition of the DES. The broadening and red-shifting of the CO band are observed in both urea and DES solutions, which are induced by the change of the distal ligands around the heme. Although the FTIR study is sensitive to structural changes in the active site, it does not provide quantitative information about structural dynamics related to the catalytic activity itself. Thus, we carried out two-dimensional IR spectroscopy of the CO mode, which suggests that there is a different conformer that could be related to the enhanced catalytic activity in DES. In particular, the spectral diffusion dynamics of that conformer exhibits quite different behavior. The experimental results lead us to propose a hypothesis that the DES increases the population of the conformer with distal ligand lysines close to the reaction center through the combining effect of urea and EAC, which results in the enhancement of the peroxidase activity of cytochrome *c*. We anticipate that the present experimental work stimulates future investigations of the effects of DES on biocatalysis.

## INTRODUCTION

Cytochrome *c* (Cyt*c*) is an electron transfer hemoprotein comprising 104 amino acids, and it plays a key role in the life-supporting synthesis of adenosine triphosphate (ATP) in the mitochondria.^1^ Interestingly, Cyt*c* is known as a multifunctional enzyme because it can adopt several different conformers with various different biological functions beyond respiration.^2,3^ It turned out that pH has a huge impact on the structure of Cyt*c*, and five distinct spectroscopic forms, states I–V, of this protein were observed with the variation of pH between pH = 1 and pH = 12.^4^ State III, the dominant one at neutral pH, is considered as the native conformation of the protein, and an alkaline conformer, state IV, is formed at pH above ∼8.5–9.5. The alkaline conformer of the Cyt*c* exhibits a weak peroxidase activity^4^ and functions as an electron transfer gate as well as a binary molecular switch where the reduction potential of the protein is strongly dependent on pH.^5^ The other proapoptotic conformer with enhanced peroxidase activity is capable of catalyzing the peroxidation of a mitochondria-specific phospholipid, cardiolipin, which is essential for the release of proapoptotic factors from the mitochondria to initiate the subsequent apoptotic processes, the formation of the apoptosome, and the progression of apoptosis.^6–9^ Recently, four phosphorylation sites on Cyt*c* were identified, which indicates that its multiple functions are regulated by cell signaling pathways.^10–12^

Consequently, the focus has been placed on Cyt*c* as an interesting heme-based biocatalyst.^13–15^ Papadopoulou *et al.*^16^ recently investigated the effect of a choline chloride and ethylammonium chloride-based deep eutectic solvent (DES) formulated with three hydrogen-bond donors (HBDs), i.e., urea, ethylene glycol, and glycerol, on the peroxidation activity of Cyt*c* and horseradish peroxidase. It is noted that DESs can be easily prepared by mixing hydrogen-bond acceptor (HBA) and hydrogen-bond donor (HBD) molecules. One of the most popular HBA molecules for DESs are ammonium salts such as choline chloride, and the commonly used HBDs include sugars, polyols, and amino acids.^17^ DESs adopt a liquid state at room temperature and are generally cheap, nontoxic, nonvolatile, nonflammable, thermally stable, and even biodegradable solvents, which make them advantageous over ionic liquids. Therefore, DESs have recently been studied as excellent solvents for various biocatalytic reactions.^18,19^ Although the presence of DES was shown to affect the peroxidase activity of Cyt*c*, it does not influence that of horseradish peroxidase, where the activity enhancement depends on the concentration of the DES and the type of ammonium salt and hydrogen-bond (H-bond) donor molecules used. Interestingly, the addition of 30% v/v ethylammonium chloride (EAC)/urea DES to the reaction mixture increases the peroxidase activity to almost 100-fold compared to that in a buffer solution. This effect was not observed when either EAC or urea was added to the Cyt*c*/buffer solution separately.

Intrigued by the highly enhanced activity of Cyt*c* peroxidase in the EAC/urea DES solutions, we herein evaluate the structure and dynamics of carbon monoxide Cyt*c* (COCyt*c*) in the buffer, DES, EAC, and urea solutions using FTIR and 2D IR spectroscopy. The aim of this research is to gain structural information around the heme domain and ultimately to understand the molecular origin of the enhanced biocatalytic activity of the peroxidase activity of the Cyt*c* induced by the DES.

## METHODS

### Materials

Cytochrome *c* from the equine heart (>95% protein content), urea, ethylammonium chloride, monobasic and dibasic potassium phosphate, sodium dithionite, and D_2_O were purchased from Sigma-Aldrich and used as received. The pH 7 potassium phosphate buffer solutions were prepared in water and D_2_O. Carbon monoxide cytochrome *c* was prepared by reduction with sodium dithionite solution, followed by purging with CO gas for ∼1 h. The detailed preparation of urea-ethylammonium chloride DES was described elsewhere.^20^ In short, EAC was mixed with urea (molar ratio of 1:1.5) and incubated at 80 °C for ∼1 h until a colorless liquid was formed. The resulting DES was mixed with phosphate buffer to achieve the desired concentrations. The cytochrome *c* concentration for FTIR and 2D-IR spectroscopy was 16 mM for buffer, EAC/buffer, and urea/buffer solutions and 11 mM for the DES/buffer solution.

### FTIR and 2D-IR spectroscopy

FTIR spectra were recorded with a Bruker VERTEX 70 spectrometer having a frequency resolution of 1 cm^−1^ at 22 °C. FTIR and 2D-IR experiments were performed with a Harrick IR sample cell consisting of two CaF_2_ windows (2 mm thickness each). A 56 *μ*m thick Teflon spacer was used to control the thickness of the solution sample.

To perform the 2D-IR experiments, a mid-IR beam centered at ∼1940 cm^−1^ with a bandwidth (FWHM) of ∼200 cm^−1^ and an energy of 800 nJ, generated in a laser setup consisting of a femtosecond Ti:sapphire oscillator (Tsunami, Spectra-Physics), a regenerative amplifier (Spitfire, Spectra-Physics), and an optical parametric amplifier (OPA-800C, Spectra-Physics), was split into three excitation beams and a local oscillator (**k**_1_, **k**_2_, **k**_3_, and **k**_LO_). The **k**_1_, **k**_2_, and **k**_3_ beams were first focused onto the sample in a boxcar geometry and then collimated by parabolic mirrors after passing through the sample. The time delay *τ* between the **k**_1_ and **k**_2_ pulses, which is the coherence time during which the excited vibrational modes in the sample solution undergo coherent oscillation, the time delay between the **k**_2_ and **k**_3_ pulses, which is the waiting time (*T*_w_), and the time delay *t* between **k**_3_ and **k**_LO_, corresponding to the detection time, were varied in the 2D-IR experiments. The phase-matched signal field emitted from the sample was combined with a local oscillator pulse (**k**_LO_) for heterodyne detection. The heterodyne-detected signal was then spectrally resolved by a monochromator and measured through a 32-element MCT (HgCdTe) array detector equipped with a high-speed data acquisition system. The 2D-IR signal *S*(*ω_t_*, *τ*; *T*_w_) was collected by scanning *τ* at a fixed *T*_w_ and subjected to numerical Fourier transformation with respect to *τ*. The rephased and nonrephased 2D-IR spectra were summed to obtain the purely absorptive 2D-IR spectrum *S*(*ω_t_*, *ω_τ_*; *T*_w_), which was finally plotted against the initial excitation frequency *ω_τ_* and the final emission frequency *ω_t_* at a given *T*_w_.

## RESULTS AND DISCUSSION

### FTIR and 2DIR spectroscopy

**FIG. 1.**
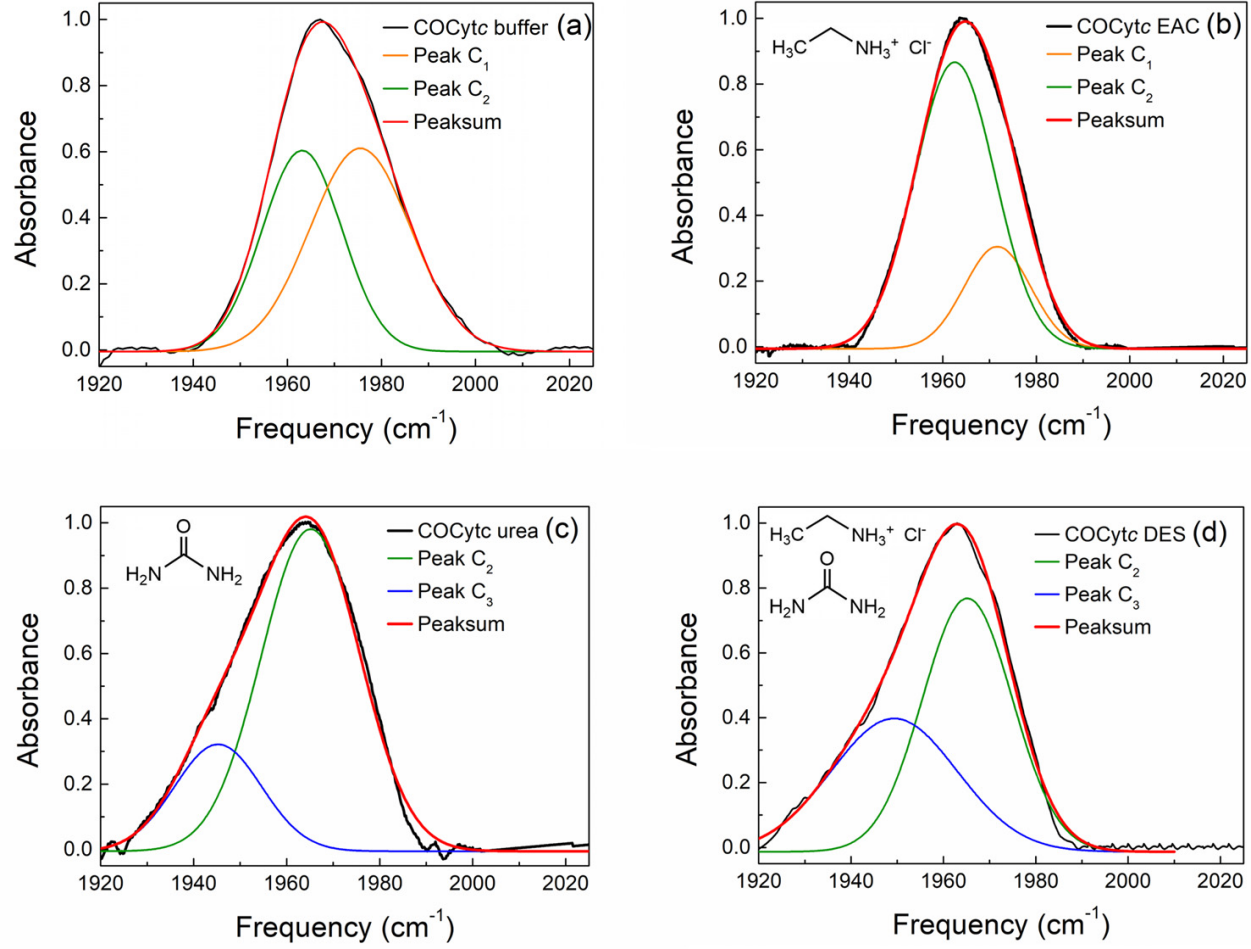
FTIR spectra of COCytc in (a) buffer, (b) EAC/buffer, (c) urea/buffer, and (d) the DES/buffer solutions. Black lines show the background-subtracted experimental data. Orange, green, and blue lines show the results of fitting to Voigt line shape functions for the C1, C2, and C3 states, respectively (see the text). Red lines show the sum of the fitted functions.

Figure 1 shows the FTIR spectra of carbon monoxide cytochrome *c* (COCytc) in buffer solution, 1.9 M EAC/buffer solution, 2.9 M urea/buffer solution, and 30% v/v DES/buffer solution. The concentrations of EAC and urea in the 30% v/v DES/buffer solution are also 1.9 M and 2.9 M, respectively. In the buffer solution [Fig. 1(a)], the CO stretch band appears at around 1970 cm^−1^ with a clear shoulder peak in the high frequency side. When the EAC is added to the buffer solution [Fig. 1(b)], the shoulder peak intensity decreases and the CO stretch band becomes significantly narrower than that in the pure buffer solution. Previously, Jaganathan *et al.* showed that the addition of ethylammonium based ionic liquid (ethylammonium nitrate) stabilizes the protein structure of Cyt*c*. They suggested that the stabilization of the Cyt*c* structure could be attributed to the electrostatic interaction between ethylammonium nitrate and the charged groups in Cyt*c*, as well as the interaction between the ethyl group of the ethylammonium cation and the hydrophobic part of Cyt*c.*^21^ The observed spectral narrowing of the CO stretch band of COCyt*c* in the EAC/buffer solution could result from the reduction of the population of COCyt*c* conformers with high CO frequency. In contrast, in the urea/buffer and DES/buffer solutions, the CO stretch band appears to be significantly broad and is red-shifted. Kim *et al.* observed a similar red-shifting and broadening of the CO stretch band upon addition of guanidium hydrochloride (GuHCl), a denaturant like urea.^22^ Thus, the observed CO peak broadening and red-shift in DES/buffer and urea/buffer solutions could result from a perturbation of the protein structure by denaturants. In all solutions, the FTIR spectra exhibit a complicated line shape, but they can be well-fitted with two Voigt functions as shown in Fig. 1. The detailed fitting results are listed in Table S1 in the supplementary material. In the case of carbon monoxide myoglobin (MbCO), another hemoprotein, which similarly to the Cyt*c* contains iron-bound heme in its structure and can bind small ligands such as O_2_, CO, and NO, the CO stretch band was used to identify different conformers that are denoted as A_0_–A_3_ differing from one another by the detailed conformations of neighboring histidines and heme-bound CO.^23,24^ In addition to the frequency shift of the CO stretch band, the intensity ratios of the involved states associated with different conformers was also found to be dependent on the solvent type, temperature, and pH.^25,26^ Therefore, we expected that the detailed FTIR analyses of the COCyt*c* CO stretch bands provide the structural information in various solvents. Based on the fitting results (Fig. 1 and Table S1), we can assign three spectroscopically distinguishable peaks of COCyt*c* as the C_1_ (∼1974 cm^−1^, observed at a higher frequency in EAC/buffer and buffer solutions), C_2_ (∼1965 cm, observed in all the solutions), and C_3_ state (∼1945 cm^−1^, observed at a lower frequency in DES/buffer and urea/buffer solutions), which are assumed to be associated with different conformers. The C_3_ conformer may be related to the catalytic activity enhancement of Cyt*c* because it is observed only in DES/buffer and urea/buffer solutions. Choi *et al.*^27^ showed that, at the A_3_ state of MbCO, the distance between the distal Histidine64 H atom and the CO oxygen atom is shorter than those of the other states, which results in a stronger H-bond between them. Such a H-bonding interaction induces an increase in the CO bond strength and a red-shift of the CO stretch band. It was further shown that the distal histidine plays an important role in the peroxidase activity of hemoprotein^28^ and that the A_3_ state is related to the expression of the peroxidase activity of double mutated myoglobin (T67R/S92D) in which the rotation of distal histidine was restricted. In the present case of COCyt*c* in a buffer solution, the CO binds to the heme iron of Cyt*c* and it replaces the bond between the Methionine (Met80) sulfur and the iron, where Met80 is the nearest ligand.^29,30^ Previous studies showed that the addition of 3 M urea induces the exchange of Met80 with another distal ligand lysines (Lys79 or Lys72/73) to ligate the Fe atom of the Cyt*c* heme, and if the concentration of urea is higher than 6 M, histidine (His33) replaces with Met80.^31,32^ Therefore, in the 2.9 M urea/buffer solution, it is expected that the Lys79 gets close to the CO instead of Met80 in Cyt*c* in the buffer solution. Thus, it is highly possible that the C_3_ state observed in our FTIR spectrum can be assigned to the conformer with H-bonding interaction between the NH_2_ of Lys79 and the oxygen atom of CO. The C_2_ state observed in the FTIR spectra of COCyt*c* in urea/buffer and DES/buffer solutions is related to the conformer with Met80 close to CO. The C_1_ and C_2_ conformers in the buffer and the EAC/buffer solutions could be associated with two different configurations of Met80 in the vicinity of the CO in COCyt*c*. It should be noted that the FTIR spectrum in the DES/buffer solution is quite similar to that of the urea/buffer solution, even though the DES/buffer solution has EAC. However, the detailed analysis through fitting shows that the relative population of the C_3_ conformer in the DES/buffer solution is larger than that in the urea/buffer solution (Table S1). This observation suggests two possible scenarios about the effect of DES on the local structure around the CO-heme in COCyt*c*. The first is that even though the EAC in DES does not introduce a significant structural change around heme, the EAC stabilizes the C_3_ conformer more effectively than the C_2_ in the DES/buffer solution. If the C_3_ state is indeed the active conformer for catalytic activity, this scenario explains the enhanced peroxidase activity by DES. The previous study suggests that, in low urea concentration (0–4 M), Met80 can be replaced by the Lys79 and Lys72/72 which are in equilibrium within the compact conformation of cytochrome *c*.^31^ In such a state, urea induces the increased dynamics of the heme region of Cyt*c* and makes substrates more accessible to the heme iron. It is possible that C_3_ corresponds to this state. The second possibility is that the EAC induces significant changes in the heme proximity structure. Like in the case of the 6 M urea,^31,32^ histidine (His33) gets close to the CO and forms a H-bond with the CO. In other words, the neighboring ligand is replaced from lysines to histidine due to the addition of EAC to the urea/buffer solution. Thus, the enhanced catalytic activity in the DES/buffer solution in this case results from the positioning of the histidine (His33) in the active site. It should be noted that Papadopoulou *et al.* measured the circular dichroism (CD) spectra of Cyt*c* in several DES/buffer solutions,^16^ and the observed spectral differences in the CD Soret region indicate that the heme plane undergoes a reorientation in the active site pocket. Such a structural change can contribute to both scenarios.

It is unfortunate that one cannot distinguish the two cases, i.e., the H-bonds between the NH group of His33 and CO and between the NH_2_ of Lys79 (or Lys72/73) and CO because they both induce a similar frequency shift of the CO stretch mode. This is why the FTIR analyses cannot provide sufficient information for explaining the structural changes around heme.

**FIG. 2.**
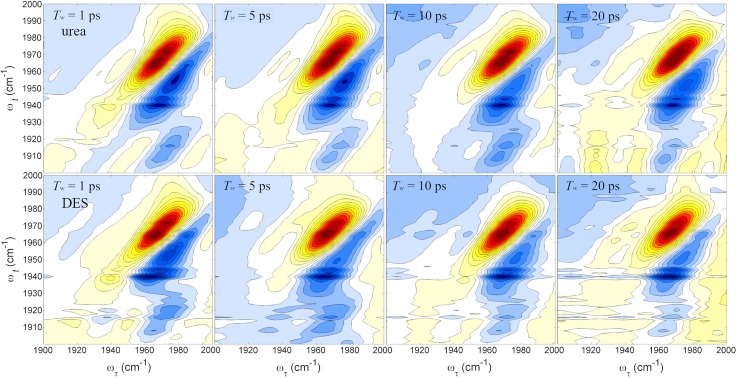
The 2D IR spectra of the carboxycytochrome *c* for chosen waiting times *T*_w_. The top panels are the spectra from the COCyt*c* in the 2.9 M urea solution, and the second row are the spectra from the COCyt*c* in a 30% v/v DES/buffer solution.

2D-IR spectroscopy, as explained elsewhere,^33,34^ has widely been used to study the structural evolutions of a variety of proteins. Briefly, 2D-IR spectroscopy can monitor picosecond time scale equilibrium dynamics by vibrationally labeling molecules or chemical groups with their initial frequencies (*ω_τ_*) and then registering the final frequencies (*ω_t_*) of the initially labeled molecules after a finite waiting time (*T*_w_). The changes in the peak shapes with increasing *T*_w_ are directly related to the structural evolution of the protein. In the present work, we use the CO stretch mode of COCyt*c* as the IR probe of the local environment around the heme group in COCyt*c*. Figure 2 shows the 2D IR vibrational echo spectra collected at four different waiting times for COCyt*c* in the urea/buffer and the DES/buffer solutions. The other two series of the 2D-IR spectra of COCyt*c* in the buffer and the EAC/buffer solutions are shown in Fig. S4 in the supplementary material. In all the 2D-IR spectra, there are two dominant peaks, where the positive (red) peaks along the diagonal (*ω_τ_* = *ω_t_*) arise from the ground state bleaching and stimulated emission contributions that involve the fundamental vibrational transition (ν = 0 ↔ ν = 1). The negative peaks (blue) below the diagonal line result from the excited state absorption from ν = 1 to ν = 2 states.^33,35^ The 2D-IR spectra of COCyt*c* show two positive peaks on the diagonal line at the same positions identified with the fitting analyses of the FTIR spectra. The excited state absorption contributions also appear as two negative peaks that are red-shifted from the fundamental frequencies along the detection frequency axis. Here, it should be mentioned that unlike MbCO, the CO ligand is difficult to be introduced into the heme pocket of Cyt*c* (see Fig. S2; supplementary material). Therefore, only a small amount of COCyt*c* could be prepared for the present experimental studies. Nonetheless, the high power and the improved sensitivity of our laser system allowed us to measure high-quality 2DIR spectra for really low-concentration COCyt*c* solutions due to the difficulty of the CO ligation to the heme reaction center. Note that the absorbance of COCyt*c* in the DES/buffer solution is as small as 0.01 and that in other solutions, it is about 0.02 after the background subtraction.

At a short waiting time of 0.2 ps, inhomogeneous line-broadening makes the 2D-IR peak diagonally elongated, which results from the interactions between the CO and the heterogeneous environments within the heme pocket. With increasing *T*_w_ up to ∼40 ps, the structural evolution causes a spectral diffusion of the transition frequency, which, in turn, results in a change in the line shape of the 2D-IR peak, i.e., rounding of the diagonal peak in the 2D-IR spectrum. As can be found in all the 2D-IR spectra in Fig. 2, a weak negative peak is down-shifted by approximately ∼20–25 cm^−1^ relative to the main negative peak. This additional negative peak originates from either the higher-order field-matter interactions, e.g., fifth-order process or the transition from the overtone state to the second overtone state due to the two-photon transition by a strong pump pulse. However, the weak negative peaks are of no importance because we focus on the spectral features of the main diagonal peaks only.

It is clear that the 2D-IR spectroscopy has an enhanced frequency resolvability compared to the linear FTIR spectroscopy. In the urea/buffer and DES/buffer solutions, diagonal peaks corresponding to the C_2_ and C_3_ states are observed separately with an almost equal intensity (Fig. 2). In the corresponding FTIR spectra [Figs. 1(c) and 1(d)], however, the high frequency peak (C_2_) appears to be more intense than the low frequency one (C_3_). This indicates that the transition dipole strength of the CO stretching mode of the C_3_ conformer is larger than that of the C_2_ state. This again supports our assignment that the C_3_ state is associated with the conformer with an H-bond between the CO oxygen atom and the NH_2_ group of lysines. This is also the case of MbCO, where the H-bonding interaction between the CO and the ligand increases the bond length of CO, which results in a frequency red-shift of the CO stretching mode and an increase in the corresponding vibrational transition dipole moment. The spectral diffusions of the two states can be extracted by analyzing the time-dependent 2D-IR spectra with the centerline slope method (CLS; see the supplementary material).^36–38^ Since there exist two conformer states for COCyt*c* in the DES/buffer and urea/buffer solutions, the CLS analyses are performed for the low and high frequency peaks separately, which provide information about the spectral diffusion dynamics of the C_3_ and C_2_ states (Fig. S5, supplementary material).

**FIG. 3.**
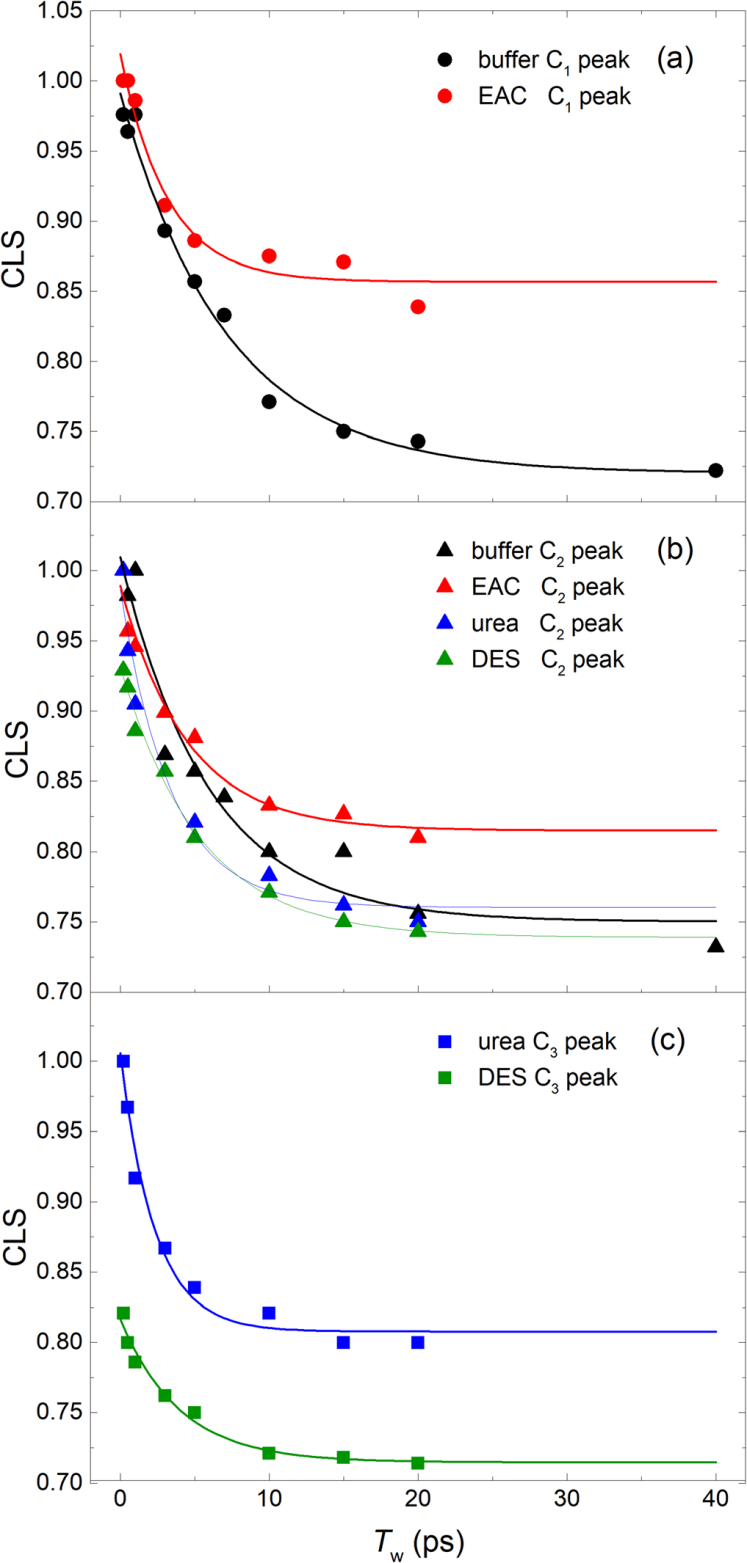
Center line slope (CLS) data (points) for the carboxycytochrome *c* in the phosphate buffer, EAC, urea, and 30% v/v DES/buffer solutions for (a) high frequency peaks in the buffer and in a 1.9 M EAC solution corresponding to the C_1_ state, (b) low frequency peaks in the buffer and in a 1.9 M EAC solution and high frequency peaks in a 2.9 M urea solution and in a 30% v/v DES/buffer solution correspond to the C_2_ state, and (c) low frequency peaks in a 2.9 M urea solution and in a 30% v/v DES/buffer solution correspond to the C_3_ state. The fits, shown as solid curves, are to an exponential decay function with an offset.

The CLS data and the exponential decay fits are shown in Fig. 3. A single exponential function with a constant offset is found to fit the CLS data well (see Table I). Figure 3(a) shows the CLS data of the C_1_ state observed in the COCytc in the buffer and EAC/buffer solutions. As listed in Table I, the CLS decay time constant becomes fast with the addition of EAC in the buffer solution. However, there is a large increase in the offset value in the EAC/buffer solution. A similar increase in the offset value is observed in the CLS data of the C_2_ state which is plotted in Fig. 3(b). Interestingly, the effect of EAC is quite different in the EAC/buffer solution and DES/buffer solution. When added to the buffer solution, EAC produces an increase in the offset value of the CLS data of the C_2_ state, which is almost the same as one in the C_1_ state. However, the EAC in the DES solution does not produce the increase in the CLS offset value in the C_2_ state. It indicates that the urea affects the structural variation around the heme pocket more directly than EAC. This difference may also be related to the eutectic properties of DESs in the aqueous solution, in which the solvent structures can uniquely change by the concentration of DESs.^39,40^ In Fig. 3(c), the CLS data and fitting results of the C_3_ states of the COCytc in DES/buffer and urea/buffer solutions are shown. The C_3_ conformer in the DES/buffer solution is likely to be related to the enhanced catalytic activity of the COCyt*c*. The CLS decay time constants of the two C_3_ states show a significant difference between urea and DES, which are 2.3–4.0 ps, respectively. The decay time for the C_2_ conformer also increases from 3.4 ps to 5.3 ps with the addition of EAC. Comparing the spectral diffusion dynamics between the C_2_ and C_3_ states in the DES/buffer and urea/buffer solutions, we found that the FFCF of the C_3_ state decays faster than that of the C_2_ state. This increased FFCF decay rate can be caused by the H-bonding interaction between lysines and CO in the C_3_ state. Another notable difference observed in Fig. 3 is that the ultrafast decay of the FFCF, which is associated with the so-called homogeneous dephasing component, depends on the presence of DES. The initial CLS values for both the C_2_ and C_3_ states of COCyt*c* in the DES/buffer solution show an appreciable deviation from unity at *T*_w_ = 0 ps, where the initial CLS value was obtained from the extrapolation of the CLS data with the exponential fit functions. The homogeneous dephasing component arises mainly from the inherently faster motion, which cannot be resolved with our approximately 100 fs time resolution.

**TABLE I.** Results of the fitting of the CLS curves. Frequency is a peak frequency of the fitted peak shown in Fig. 1. y_0_ is the offset and T is the decay time obtained by fitting data with a single exponential function.

	Frequency (cm^−1^)	*y*_0_	Τ (ps)
Buffer	1975.5	0.72	7.1
	1963.1	0.75	5.9
EAC	1973.3	0.86	3.2
	1962.6	0.82	4.4
Urea	1965.2	0.76	3.4
	1945.3	0.81	2.3
DES	1965.2	0.74	5.3
	1949.4	0.71	4.0

The observed differences in the FFCF decay times depending on solvents may be attributed to their viscosities, even though the structural fluctuations of folded proteins were shown to be weakly dependent on the solvent viscosity.^41,42^ Considering the large difference in the viscosities of the urea/buffer and the DES/buffer solutions, such macroscopic solvent property might be of use for explaining the increase in the spectral diffusion time constants in DES. However, the viscosity dependence of the structural fluctuations cannot be the source of the increased homogeneous component observed for different conformers of COCyt*c* in the DES/buffer solution.

The 2D-IR spectra of COCyt*c* in the EAC/buffer and the buffer solution were also measured (Fig. S3), and the spectral diffusion dynamics were analyzed to understand the effect of EAC on the dynamics of COCyt*c* [Figs. 3(a) and 3(b)]. As shown in Table I, the FFCF decay rate becomes fast with the addition of EAC to the buffer solution. These results indicate that the viscosity increase cannot explain the slow-down of the spectral diffusion dynamics in the DES/buffer solution, because the EAC also increases the solvent viscosity but simultaneously increases the FFCF decay rate. From the comparisons between the experimental results shown in Fig. 3 and summarized in Table I, EAC causes a significant increase in the offset value, which is related to static inhomogeneity. This experimental observation is consistent with the previous work showing that the structural stabilization of the Cyt*c* protein by ethylammonium based ionic liquids like EAC is caused by the direct binding of ionic liquids with Cyt*c*.^21^ The binding of EAC to the COCyt*c* can increase the static inhomogeneity, which makes the FFCF approach to an offset constant within the experimental time window. However, the fast CLS decay rate observed in the EAC/buffer solution indicates that the same structural stabilization effect by EAC molecules does not impact on the local dynamics of amino-acid residues close to the heme buried inside the protein. In relation to this, Finkelstein *et al.* measured the FTIR and 2D-IR spectra of the CO ligated Horseradish peroxidase (HRP-CO) with and without the substrate benzohydroxamic acid (BHA).^43^ They showed that the presence of BHA causes the corresponding CO stretch IR spectrum to be a single peak, which is in contrast to the doublet spectrum of HRP-CO in the buffer solution without BHA. Furthermore, the decay of the FFCF for the BHA-bound HRP is much faster than the free HRP, but the amplitude of decay is smaller within the 2D-IR time window (>40 ps). The results indicate that the BHA binding significantly reduces the ultrafast protein fluctuations within the 2D-IR time window. Their experimental observation that the substrate binding to HRP dynamically restrains the structural flexibility of HPR-CO is quite similar to ours for the EAC solution in which the line shape of the corresponding FTIR spectrum becomes narrow and the CLS data suggest an increased structural heterogeneity due to the increase in potential energy barriers between different conformers.

In summary, we here propose that the C_3_ state is associated with the catalytically active conformer that is related to the enhanced peroxidase activity. Furthermore, from the present IR spectroscopic studies of the effects of urea and EAC in the COCyt*c* DES/buffer solution, we found that the 2.9 M urea increases the population of the C_3_ state that has lysines close to the heme. In the COCyt*c* protein, the H-bonding interactions between CO and NH_2_ in lysines cause a frequency red-shift of the CO stretch mode in the C_3_ conformer. Second, the added EAC molecules increase the population of the C_3_ conformer by their binding to COCyt*c* without causing any significant change of the heme structure. Now, it becomes clear that the spectral diffusion dynamics of IR probe frequency, which can only be extracted from time-resolved 2D-IR spectroscopy, provides direct information about structural dynamics within the enzyme active site as well as the role of solvents in protein functions.

## CONCLUSIONS

The effect of ethylammonium chloride/urea DES on the local environment around the heme site of Cyt*c* has been studied by using FTIR and 2D-IR spectroscopy of the CO stretching mode of COCyt*c*. The addition of EAC induces both a frequency red-shift of the CO stretch mode and narrowing of the band, which results from the protein structure stabilization due to the direct interaction between EAC and Cyt*c*. On the other hand, the addition of urea and DES to the Cyt*c* solution induces a much larger frequency red-shift and notable broadening of the CO stretch band, which can be attributed to the change of the distal ligand directly interacting with the CO bound to the heme of Cyt*c*. The *T*_w_-dependent line shape analyses of the 2D-IR spectra provide information about the structural fluctuation dynamics of COCyt*c* in urea/buffer and DES/buffer solutions. From the analyses of the FTIR and 2D-IR results, we conclude that the most plausible hypothesis is that the C_3_ conformer is the active conformer for the peroxidase reaction with lysines close to the heme pocket and that the urea increases the population of this conformer and the EAC molecules in the DES further stabilize it, which results in the enormous enhancement of the catalytic activity of the Cyt*c*. To confirm the hypothesis proposed here using the experimental results obtained with FTIR and 2D-IR spectroscopic methods, detailed molecular dynamics simulation studies in the future would be necessary.

## SUPPLEMENTARY MATERIAL

See the supplementary material for the details on FTIR analysis, UV-vis and CD spectroscopy, and CLS analysis.
